# Assessing through a longitudinal study of dietary habits among Romanian school children: effects of COVID-19 pandemic as well as of a school based educational program for promotion of healthy nutrition

**DOI:** 10.1007/s00394-024-03492-x

**Published:** 2024-09-17

**Authors:** Anda-Valentina Trandafir, Lucia Maria Lotrean

**Affiliations:** 1 Department of Community Medicine, Iuliu Hatieganu University of Medicine and Pharmacy, Cluj-Napoca, Romania; 2 Research Center in Preventive Medicine, Health Promotion and Sustainable Development, Iuliu Hatieganu University of Medicine and Pharmacy, Cluj-Napoca, Romania

**Keywords:** COVID-19 pandemic, Health education, Children, Dietary habits, Self-perceived changes

## Abstract

**Purpose:**

The outbreak of COVID-19 has forced implementation of safety measures, leading to changes in people’s lives. This study investigated several dietary habits among Romanian children before and during the pandemic. Also, it assessed the effectiveness of an educational program promoting healthy diet and evaluated factors influencing certain eating habits.

**Methods:**

A longitudinal study was conducted in schools. Students were assigned to Control and Intervention groups. Data were collected at baseline (T1) (October -November 2019, 880 children) and post-intervention (T2) (December 2020-February 2021, 484 students). 350 children participated in both assessments.

**Results:**

Baseline measurements revealed inadequate consumptions of fruits, vegetables, dairy products, and increased intake of soft drinks and sweets. At follow-up, the Intervention showed significant improvements on average intake of fruits and vegetables and in children eating ≥ 5 servings/day, compared to its baseline. Regarding self-perceptions on dietary habits during confinement, children from both groups reported healthier behaviors, with a significant decrease of comfort food. Compared to the Control group, Intervention increased its average intake of fruits and vegetables, consumed more ≥ 5 portions/day, reduced the number ≥ 2 cups/day of carbonated beverages and ≥ 3 portions/day of sweets. Age, gender, weight management, body mass index were associated with dietary habits at T1. Age, gender, Intervention group, weight management influenced eating patterns at T2. Eating behaviors at T1 influenced dietary habits at T2.

**Conclusion:**

Our program demonstrated positive changes in students’ dietary habits, despite challenges of COVID-19. Results highlight the role of health education and emphasize the importance of integrating such programs consistently in schools.

## Introduction

Since December 2019, the world has gone through a life-changing challenge with the onset of COVID-19 pandemic [1]. Due to the high number of cases of severe acute respiratory syndrome caused by the SARS-CoV-2, on 30 January 2020, the World Health Organization (WHO) declared the outbreak a Public Health Emergency of International Concern [2] and on 11 March a global pandemic [3]. In Romania, the first case was registered on 26th of February and by March 26, the number of confirmed cases exceeded 1000 sick people [4].

From the earliest stages of COVID-19 pandemic, many governments have implemented countermeasures to control viral exposure and contagion. Quarantine maneuvers were adopted, such as travel bans, social distancing, home confinement, closedown of schools, universities and other public spaces, military curfews, wearing a face mask [5]. People were forced to stay at home, restricting their mobility only for essential and urgent reasons, such as shopping for food, buying medicines, work situations and seeking medical help [3, 6, 7]. Most residents started working from home, while students continued their schooling by learning virtually.

Of those affected by the aforementioned changes, youth represent a vulnerable population because they are going through a period of development. In addition to the physical changes, children also cross a psychological stage and are willing to gain more and more independence. Typically, after reaching the age of 12, adolescents begin to have more autonomy in making their own food choices and during this period the parental control over their eating behaviors tends to diminish, while the influence of peer-pressure increases [8, 9]. If not carefully monitored, children are susceptible to unhealthy behaviors, such as smoking, poor eating habits towards unhealthy food and sugary beverages and poor exercise [10–13] with a lifelong impact if they are sustained over time. Once adopted, these patterns are difficult to change and increase the risk of obesity and non-communicable diseases in adulthood [7, 14, 15]. Therefore, it is crucial to prioritize early intervention in promoting healthier dietary patterns among children, as it is more effective in preventing negative outcomes rather than attempting to reverse them later on [16, 17].

There are several recommendations for healthy nutrition among children.

- a daily intake of fruit and vegetables of at least 400 g, representing five servings a day [18, 19], (each serving contains around 80 g)
- consumption of 2–3 servings of dairy products depending on children’s age (one serving is represented by one cup of 250 ml of milk or yoghurt for instance), being recommended to be included in the diet low fat dairy products [18].
- consumption of added sugar should provide no more than 10% of the total daily energy intake, hence a limited amount of sweetened beverages and sweets should be consumed per week [20].

Data from several countries show the fact that children eat insufficient fruits and vegetables and too much processed foods, such as sugary beverages and sweets [21]. While fruits and vegetables are rich in vitamins, fibers and other bioactive compounds [22], sugar sweetened drinks contain large amounts of sugar [21], which make them have little nutritive benefits [23–25]. Carbonated drinks are among the most consumed such drinks among teenagers [26], while sweets place among the favourite snacks [27]. These explain why added sugars are widely present in children’s diets [28, 29]. Along with fruit juices and confectionery, soft drinks and sweets are the main contributors of added sugar intake [28]. In the US, sweetened beverages represented 47% of total added sugars in children [30], while baked goods and sweet snacks accounted for 21–31% [31, 32]. High reliance on sugary items, can have harmful effects on health: great amounts of added sugars have been found to increase the risk of obesity, dyslipidemia, type 2 diabetes, hypertension and cardiovascular disease [33]. Moreover, the literature suggests a positive correlation between sugars and unhealthy behaviors: watching TV or spending at least an hour in front of screens have been associated with increased consumption of sweets and with regular consumption of carbonated drinks [34, 35], while “eating on the go” with high a prevalence of sweets intake [36].

Overweight and obesity have reached increasing levels among young people globally. In 2008, the prevalence of overweight and obesity was nearly 25% in children in Europe and by 2010, it had increased to 33% [37, 38]. These findings highlight the impact of a poor qualitative diet in the aetiology of obesity where eating behaviors are among factors that can be changed [39].

Schools are an ideal environment for cultivating healthy nutritious practices among children, as they encompass the majority of them, including those who lack positive modelling at home [40–42]. Given that children spend a significant amount of time in classrooms, schools represent a suitable arena for implementing educational intervention programs aimed at fostering healthy nutrition [43, 44]. A systematic review conducted by Mari W Murimi et al. on papers published between 2009 and 2016 revealed that factors to the success of an education program consisted in interventions lasting longer than 6 months along with a multicomponent approach: practical activities such as cooking demonstrations, gaming or gardening, parental engagement and targeted dietary patterns to be changed [45]. The Pro Children study implemented in three European countries, carefully designed following a comprehensive theoretical protocol, showed significant improvements at one year follow-up in the intervention group compared to the control group, with a notable increase of 25% of children eating more fruits and vegetables [40, 46].

Furthermore, a systematic review revealed that interventions promoting dairy consumption among school aged children proved successful in increasing dairy intake [47]. Dairy products are an important source of micronutrients (calcium, vitamin B2, vitamin B12) essential in children’s growth [48]. However, despite their substantial role in children’s development, extensive literature highlighted a negative trend in the consumption of dairy products in the young population, especially in adolescents [49, 50]. This problem is explained, at least in part, by the fact that most interventions targeting dairy products intake were not directed at dairy products alone, but were part of a larger program to promote healthy eating behaviors [47, 51].

According to the latest report before the pandemic, the situation in Romania was not far from that in Europe, where most young people did not adhere to the nutrition specialists’ recommendations. The report from 2017/2018 showed that nearly half of students did not include fruits and vegetables in their daily diet. Boys were less likely to eat fruits and vegetables. The prevalence of children not consuming fruits and vegetables every day increased with age, reaching 57% for 11-year-olds and 60% for 15-year-olds. As for daily sweets consumption, Romania ranked among the leading European countries. Unlike younger children, adolescents increased their daily intake, and there was a gender difference too, with boys surpassing girls (41% of 15-year-old boys compared to 31% of 15-year-old girls, and 37% of 11-year-old boys compared to 33% of 11-year-old girls). Similar patterns were observed in the consumption of sweetened beverages, with boys and older adolescents outnumbering girls and young teenagers and one in five young individuals consuming them daily [52, 53]. Despite these statistics, there is insufficient data available in Romania concerning the assessment of educational activities aimed at promoting a healthy lifestyle among children.

Hence, this study had three objectives. The first objective was to assess several dietary habits before and during the COVID-19 pandemic among Romanian school children. Secondly, our research evaluated the effectiveness of a school based educational program for promotion of healthy nutrition implemented shortly before the pandemics among elementary school students. Last but not least, we identified factors that influenced specific eating behaviors.

## Materials and methods

### Study design and sample

The current study is a longitudinal study which was carried out among 7 schools of two counties of Romania (Cluj and Alba). All procedures were in accordance with the guidelines of Declaration of Helsinki and were approved by Ethics Commission of „Iuliu Hațieganu” Medicine and Pharmacy University, Cluj-Napoca, Romania (134/6.05.2019).

Eight schools from two counties of Romania were invited to enroll in the study. The directors of schools were contacted and informed about the objectives and the protocol of the research, which implied that participation would entail a random assignment to either the experimental or control group. Of these, 7 school principals voluntarily agreed to participate in the study and only one refused. The principals provided the number of the 5th to 8th classes that could participate. Before the enrolment, all parents/guardians received letters with the description of the project and the purpose of the study, and they signed an informed consent for their child’s participation.

Afterwards, the research team randomly sampled the participating schools from each county, 5 into the intervention group (I) consisted of 510 children and 2 into the control group (C) with 370 pupils [54].

There were several nutrition and lifestyle related behaviors addressed by the study and the interventional program, but one main focus was on nutrition education for improving consumption of fruits and vegetables. We ran a sample size calculation in order to determine the adequate sample size before starting our research [55]. We set the level of significance 0.05 and 80% power, with a 7% difference at follow-up in the percentage of children who consumed minimum 5 portions of fruits and vegetables per day as indicated by guidelines (2% among the Control group and 9% among the Intervention group). Results indicated a minimum of 326 participants (163 in the experimental group and 163 in the Control group). However, due to expected drop-outs at follow-up as well intention to educate more children with regard to healthy nutrition, we decided to enroll all students with informed consent and present on the day of the assessment, in the study.

### Procedure

The study was conducted in 4 phases: (1) a baseline evaluation (T1), followed by (2) the implementation in the intervention group of educational activities for promotion of healthy nutrition and active lifestyle, (3) a second evaluation (T2) among all students that assessed the influences of the program on knowledge, attitudes and behaviors among children regarding diet and the physical activity, as well as self-reported behaviors of students during pandemic and finally (4), implementation of the program in the control group.

The baseline assessment was performed during school activities via questionnaires, between October 2019-November 2019. Questionnaire was administrated by the research team. Teachers were not involved in data collection although they were present during evaluation. Before completing, pupils were explained both orally by a member of the research team, and in written, at the beginning of the questionnaire, that their participation was voluntary and they were assured about the confidentiality of their answers. Children wrote their names on an envelope provided by the research team and included their questionnaire in it. By filling in the questionnaire children gave their informed consent on participation. No refusal was recorded, but there were children whose parents agreed on their participation but were absent from school the day the assessment was performed.

Starting with March 2020 with introduction of confinement measurements decreed by the Government and until the end of the school year, all the school activities were carried out online. Due to these circumstances, with the consent of the headteachers of the schools enrolled in the study, children were invited to participate in the second evaluation, through an online survey. The questionnaire was administrated in both the Intervention group and the Control group, between December 2020-February 2021. In Romania, the education system includes four levels of education: primary school (children from 1st to 4th grade), gymnasium (5th to 8th grade), high school (students in grades 9th to 12th) and university. According to the school calendar, the teaching activities are structured during September-June. Students who were in 8th grade at the first assessment were lost at T2.

The second survey was constructed using Google Form. It was initially shared to teachers on social media platforms and, with their help, to children through chat groups. Teachers were asked to remind students periodically to complete the questionnaire. Responders were fully informed about the confidentiality of their answers. Participants completed personal information, including school, gender and names for the subsequent analyses. Once the questionnaire was submitted, the collected data were automatically linked to an Excel sheet that was downloaded by the research team. Duplicate answers were excluded.

### Instruments for data collection

#### Questionnaires

The study used questionnaires that were developed based on literature data and previous questionnaires developed and tested in several studies in Romania [56, 57]. For instance, one of these studies assessed fruits and vegetables consumption among Romanian children and proved a good test-retest reliability of the questionnaire as well as the fact that the children did not express concerns with regard to the content and clarity of the questionnaire items [57].

#### Dietary data

The present study focused on the frequency of consumption of fruits and vegetables, dairy products, soft drinks and sweets during the last week (Never to 7 days). Children were also asked to self-recall the number of portions of each product they consumed per day. Responses varied from “One portion” to “More than 5 portions”, except for soft drinks, where answers varied from “Less than one cup” to “More than 5 cups”. One portion was defined as such:

- For fruits and vegetables, one portion referred to half a cup of raw or cooked vegetables, or a medium-sized fruit (an apple, a pear, an orange, etc.) or three-quarters cup of natural vegetable or fruit juice (one cup having around 240–250 ml).
- For dairy products, one serving referred to one cup of milk, or a cup of yogurt, a piece of cheese or curd the size of a box of matches, or two tbs (one cup having around 240–250 ml).
- For soft drinks, a portion was measured in cups (one cup having around 240–250 ml).
- For sweets, a portion was defined as a cake, a chocolate, wafers or candies that have a total size of two fingers (around 30 g), or a tablespoon of sugar or jam.

The portions were resampled, according to the average consumption, into categories as follows: for vegetables and fruits, “<3 portions/day”, “≥ 3 and < 5 portions/day” “≥ 5 portions/day”, for dairy products “< 2 portions/day”, “2–3 portions/day”, “>3 portions/day”, for sweets and soft drinks “< 1 portion”, “1-1.9 portions/day”, “2-2.9 portions/day”, “3-3.9 portions/day”, “4-4.9 portions/day”, “≥ 5 portions/day”.

Participants were asked about their intentions to consume fruits and vegetables and dairy products next month, with three possible responses: “Same quantity”, “A smaller quantity”, “A larger quantity”.

Also, children were asked whether they tried to choose dairy products (milk, yogurt, cheese) with a lower fat content the week before, from Monday to Sunday inclusive. (Yes/No/I don’t know).

#### Self-reported eating habits during COVID-19 pandemic

The second assessment included specific questions related to changes during the COVID-19 pandemic with regard to self-reported food intake. To avoid confusions, questions referred to specific time periods: before remote education (January-February 2020) and during remote education period (previous month). The current study investigated children’ self-assessment on their diet intake during pandemic compared to pre-pandemic period. Questions referred to the main food groups: fast food, home cooked food, purchased cooked food, potatoes, fresh fruits and vegetables, frozen or canned fruits and vegetables, dairy products, meat, processed meat, sweets, chips and snacks, soft drinks, energy drinks, rice and pasta, bread. Possible answers were “My intake is similar”, “My intake increased”, “My intake decreased” and “I don’t know”.

### School based educational program

During the pre- and peri-lockdown measures, children participated on activities for promotion of healthy nutrition and active lifestyle which were previously piloted within the Stivia program among children with hearing impairment [58]. Nine educational sessions were delivered to the children in the intervention group at school both by the teachers and the members of the research team. The sessions took place every 1 to 3 weeks and lasted 50 min each.

Topics included the importance of physical activity, general principles of hand hygiene, water hydration, the role of different food groups in a healthy diet, especially the intake of fruits and vegetables, as well as the importance of regular meals and food safety issues. Each educational session was based on a specially prepared video containing the main information in line with the theme and educational objectives. Moreover, the educational sessions included an interactive part, in the forms of games, discussions, competitions, hands-on-activities, fruit and vegetable tastings, low fat dairy products tasting, through which the key messages were reinforced. Teachers received supportive and printed materials, such as DVDs with the recorded sessions, brochures and posters. The recorded sessions assisted teachers in promotion of healthy eating behavior and an active lifestyle among students. Several posters illustrating hand washing techniques, recommendations on water and fruit and vegetable consumption, as well as a “healthy plate” with the main types of food to be served during a meal and key messages regarding food safety were displayed in classrooms and throughout the school facilities. Additionally, we distributed student’s book to children, containing information about the main topics discussed in class, accompanied by representative images and small activities, as well as home works and exercises for monitoring food consumption throughout a week, while for parents we distributed newsletters. In this way, on one hand, we aimed to stimulate discussions within the family about healthy eating behaviors, and on the other hand, to inform parents about the topics covered in this program so that they could potentially assist or encourage the implementation of these habits at home.

### Statistical analysis

Questionnaires received an identification code for the purpose of data linkage. Researchers substituted children’s names with these codes before entering data.

In order to reach out our first objective, we performed descriptive analysis of the participants sociodemographic characteristics, as well as prevalence (N %) of children’s dietary intake from T1 and T2. We also determined the average food intake using the formula: (A X B)/7, where A represents the frequency (in days) and B the number of servings per day.

Average scores were expressed as means and standard deviations (STD). Differences between T2 vs. T1 in both Intervention and Control groups were examined using Dependent t test (for continuous variables) and Chi-test (for categorial variables).

In order to investigate the impact of the program on healthy nutrition we evaluated disparities between Intervention and Control groups at T1, respectively T2, using Independent t test (for continuous variables) and Chi-test (for categorical variable).

We also investigated factors associated with the consumption of vegetables and fruits, dairy products, carbonated drinks and sweets through linear regression analyses using the following approach. At T1 the dependent variables were the average intake of each food item, whereas the independent variables considered were: age, gender (Male = 1, Female = 0), participants (Intervention = 1, Control = 0), children’s weight management (Attempts to lose weight coded as Yes = 1, No = 0 and Attempts to gain weight coded as Yes = 1, No = 0) and BMI (Body Mass Index), calculated in a previous study [54] .

Similarly, we examined factors associated with eating behaviors at T2. The dependent variables were the average intake of fruits and vegetables, of dairy products, of carbonated drinks and of sweets measured at T2, whereas the independent variables included were: age, gender (Male = 1, Female = 0), participants (Intervention = 1, Control = 0), children’s weight management (Attempts to lose weight coded as Yes = 1, No = 0 and Attempts to gain weight coded as Yes = 1, No = 0) measured at T2.

Additionally, we investigated predictive factors for dietary habits in children over the longitudinal study. The dependent variables were the average intake of each food item measured at T2 while the independents variables considered were all measured at T1 and included: age, gender (Male = 1, Female = 0), participants (Intervention = 1, Control = 0), children’s weight management (Attempts to lose weight coded as Yes = 1, No = 0 and Attempts to gain weight coded as Yes = 1, No = 0), the average intake of fruits and vegetables, of dairy products, of carbonated drinks and sweets.

We estimated standardized coefficient (ß) and 95% confidence interval (95% CI). The significance level was set to *p* < 0.05.

Data analyses was performed using IBM SPSS Statistics 26 (IBM Corporation, Armonk, NY, USA).

## Results

### Characteristics of the study sample

At T1 the sample consisted of 880 children of whom 510 were part of the experimental group and 310 in the Control group. 484 children participated in the second evaluation (300 in the Intervention group and 184 in the Control group). Out of these, 350 pupils took part in both assessments, with 208 in the Intervention group and 142 in the Control group.

### Dietary habits before COVID-19 pandemic

Table 1 shows baseline and follow-up data on the two samples of children (Intervention and Control groups) in terms of dietary patterns.

**Table 1:** Dietary habits of the whole sample of children participating at baseline (*N* = 880) and in second evaluation (*N* = 484), as well as differences in baseline characteristics between intervention and control groups (I₁ vs. C₁)

	Percentage or Mean (± STD)				
	Baseline (T1)		T_2_ (12–14 months after intervention)		
	Intervention group *N* = 510	Control group *N* = 370	Intervention group *N* = 300	Control group *N* = 184	*p*-Value
**Average consumption of vegetables and fruits (portions)**	1.57 (STD 1.25)	1.53 (STD 1.23)	1.75 (STD 1.23)	1.51 (STD 0.96)	0.699^1^
**Vegetables and fruits intake (days/week)** Less than 1 day/week 1–2 days/week 3–4 days/week 5–6 days/week	1.4 16.5 28.6 26.6 26.9	1.1 13.3 33.2 31.6 20.8	1.7 12.3 30.0 30.3 25.7	3.3 4.9 36.4 31.5 23.9	
Everyday					
**Vegetables and fruits/day** **(according to the average consumption of vegetables and fruits)**					
< 3 portions ≥ 3 and < 5 portions ≥ 5 portions	83.7 13.5 2.8	87.8 8.7 3.5	84.7 10.7 4.6	89.7 9.2 1.1	0.087^2^ 0.514^3^
**Intentions to eat vegetables and fruits next month**					
Same quantity Smaller quantity Larger quantity	58.5 8.8 32.7	66.5 5.4 28.1	61.7 5.3 33.0	62.0 7.0 31.0	
**Average consumption of dairy products (portions)**	1.27 (STD 1.29)	1.21 (STD 1.28)	1.26 (STD 1.00)	1.20 (STD 1.12)	0.507^1^
**Dairy products intake (days/week)**					
Less than 1 day/week 1–2 days/week 3–4 days/week 5–6 days/week Everyday	6.7 22.8 33.5 17.7 19.3	6.5 21.2 34.0 20.5 17.8	6.7 17.3 33.0 27.4 15.6	5.4 22.2 33.6 16.5 22.3	
**Dairy products/day** **(according to the average consumption of dairy products/day)**					
< 2 portions	78.6	80.5	77.7	78.8	
2–3 portions > 3 portions	14.3 7.1	12.7 6.8	18.0 4.3	15.2 6.0	0.488^4^
**Intentions to eat dairy products next month**					
Same quantity Smaller quantity Larger quantity	68.6 18.4 13.0	72.7 12.7 14.6	78.3 6.0 15.7	78.8 10.3 10.9	
**Low fat dairy consumption last week**					
Yes	46.3	40.8	49.3	39.1	
No I don’t know	32.5 21.2	37.6 21.6	32.3 18.4	33.2 27.7	0.082^5^
**Average consumption of soft drinks (cups)**	0.83 (STD 1.27)	0.87 (STD 1.33)	0.76 (STD 1.13)	0.82 (STD 1.28)	0.668^1^
**Soft drinks consumption (days/week)**					
Less than 1 day/week 1–2 days/week 3–4 days/week 5–6 days/week Everyday	20.4 38.5 24.7 8.8 7.6	21.9 40.8 18.9 9.2 9.2	23.7 38.7 26.0 5.3 6.3	29.3 35.4 20.7 6.6 8.0	
**Soft drinks/day** **(according to the average consumption of soft drinks/day )**					
< 1 cup 1-1.9 cups 2-2.9 cups 3-3.9 cups 4-4.9 cups ≥ 5 cups	75.3 13.1 3.9 1.6 1.8 4.3	75.1 11.4 4.9 2.7 2.2 3.8	77.3 12.0 5.3 2.0 0.7 2.7	74.5 11.4 7.6 2.2 1.1 3.2	0.956^6^ 0.387^7^ 0.590^8^ 0.935^9^
**Average consumption of sweets (portions)**	1.17 (STD 1.24)	1.17 (STD 1.31)	0.98 (STD 1.10)	1.05 (STD 1.12)	0.977^1^
**Sweets consumption (days/week)**					
Less than 1 day/week 1–2 days/week 3–4 days/week 5–6 days/week Everyday	5.7 32.0 33.9 14.5 13.9	3.5 33.0 33.5 16.2 13.8	8.0 32.0 39.6 13.4 7.0	6.0 34.3 30.4 17.9 11.4	
**Sweets/day (according to the average consumption of sweets/day )**					
< 1 portion 1-1.9 portions 2-2.9 portions 3-3.9 portions 4-4.9 portions ≥ 5 portions	58.6 22.2 9.4 4.9 1.6 3.3	61.1 20.5 8.1 4.3 1.6 4.4	64.7 23.7 8.0 0.3 0.6 2.7	63.0 22.8 8.2 2.7 0.6 2.7	0.463^10^ 0.753^11^ 0.819^12^ 0.496^13^

Note: Differences between I₁ vs. C₁ were calculated using Independent t test for continuous variables and Chi-test for categorical variables. ¹ Average consumption. ² <3 vs. ≥ 3 portions of vegetables and fruits; ^3^ <5 vs. ≥ 5 portions of vegetables and fruits. ^4^ <2 portions vs. ≥ 2 portions of dairy products. ^5^ Low fat dairy consumption last week, Yes vs. No. ^6^ <1 vs. ≥ 1 cups of soft drinks ; ^7^ <2 vs. ≥ 2 cups of soft drinks; ^8^ <3 vs. ≥ 3 cups of soft drinks; ^9^ <4 vs. ≥ 4 cups of soft drinks. ^10^ <1 vs. ≥ 1 portions of sweets; ^11^ <2 vs. ≥ 2 portions of sweets. ; ^12^ <3 vs. ≥ 3 portions of sweets; ^13^ <4 vs. ≥ 4 portions of sweets

At T1, in both samples, majority of children ate vegetables and fruits at least 3 days/weeks, but only 26.9% in the Intervention group, respectively 20.8% in the Control group, ate daily. In both groups, most children consumed less than three servings per day, and 2.8%, respectively 3.5% consumed at least five portions per day. The average consumption of vegetables and fruits was 1.57 portions/day (STD 1.25) in the Intervention sample and 1.53 portions/day (STD 1.23) in the Control sample. Moreover, more than half of them intended to eat the same amount in the following month, and about a third of them to eat a larger amount.

Similarly, in the majority of cases, children consumed dairy products at least 3 days/week, but less than a quarter in both groups consumed them daily. Majority of children ate less than 2 portions/day and less than a fifth of them (14.3% in I_1_ respectively 12.7% in C_1_) ate 2–3 servings/day. The average consumption of dairy products was 1.27 portions/day (STD 1.29) in the Intervention sample and 1.21 (STD 1.28) in the Control group. Also, more than half of children intended to consume the same amount in the following month and about 4 in 10 students in both groups stated they consumed low fat dairy products in the previous week.

Regarding soft drinks, more than half of participants reported drinking between 1 and 4 days/week, from whom 7.6% in the Intervention group and 9.2% in the Control group consumed them daily. Around a quarter of children in both groups drank more than 1 cup of beverages/day. The average soft drinks intake was 0.83 cups/day (STD 1.27) in the Intervention group, and 0.87 portions/day (STD 1.33) in the Control group.

Similarly, regarding sweets intake, more than half of participants reported eating between 1 and 4 days/week, while 13.9%, respectively 13.8% of them consumed them daily. Around 40% of them in both groups served more than 1 portions of sweets/day. The average sweets intake was 1.17 portions/day in both groups.

### Dietary habits during COVID-19 pandemic

Table 1 also presents children’s dietary patterns at the second evaluation. The majority of children consumed fruits and vegetables at least three days per week, but pupils in the Control group outnumbered pupils in Intervention group (91.8% respectively 86%). In both samples, about a quarter of children consumed them daily. More than 80% of children consumed less than three portions of fruits and vegetables per day, while a small percentage (4.6% in the experimental group, respectively 1.1% in the Control group) consumed at least five portions/day. The average intake of fruits and vegetables was 1.75 portions/day (STD 1.23) in the Intervention group and 1.51 (STD 0.96) in the Control group. Similar to the baseline assessment, 61.7% participants of the Intervention group and 62.0% of the Control group intended to maintain the number of fruits and vegetables in the following month, while a third of them aimed to increase their intake (33% respectively 31%).

Regarding dairy products, three quarters of children in the Intervention group consumed them at least three days per week, whereas the Control group consumed in 72.4% cases. Moreover, more than three quarters in both samples consumed less than two portions per day, whereas 18.0% of them served 2–3 portions/day in the Intervention group and 15.2% in the Control group. The average intake was 1.26 (STD 1.00) at I_2_ and 1.20 (STD 1.12) at C_2_. The majority intended to maintain the amount of dairy product in the following month, whereas nearly half of children in the Intervention group and nearly 40% in the Control group used low-fat dairy products in the previous week.

More than half of students in both groups declared they consumed soft drinks between one to four days per week. Among the participants, about a quarter of them consumed at least one cup of soft drinks per day. The average intake of soft drinks was 0.76 cups/day (STD 1.13) in the Intervention group and 0.82 (STD 1.28) in the Control group.

Regarding sweets consumption, majority of children ate between one to four days/week, whereas over 30% of them at least 1 portions/day. The average intake of sweets in the Intervention group was 0.98 (STD 1.10) and 1.05 (STD 1.12) in the Control group.

Table 2 presents changes in dietary behaviors from baseline to follow-up for students who participated in both assessments. Before and during the confinement, the intake of vegetables and fruits in both samples was in majority of cases at least 3 days/week and less than 3 portions/day. In addition, more than half of students in the Intervention and Control groups intended to eat the same amount the following month, whereas a third of them intended to eat a larger quantity of fruits and vegetables.

**Table 2:** Dietary habits before (baseline evaluation) and during the COVID-19 pandemic (second evaluation) in children participating in both evaluations, and characteristics differences between Intervention and Control Group (*N* = 350)

	Percentage or Mean (± STD)						
	Baseline (T1)		T_2_ (12–14 months after intervention)		*p*-Value^a^, *		*p*-Value^a^, **
	Intervention group *N* = 208	Control group *N* = 142	Intervention group *N* = 208	Control group *N* = 142	I₂ vs. I₁ *N* = 208	C₂ vs. C₁ *N* = 142	I₂ vs. C₂ *N* = 350
**Average consumption of vegetables and fruits (portions)**	1.52 (STD 1.16)	1.46 (STD 1.11)	1.84 (STD 1.31)	1.45 (STD 0.92)	**0.003** ^**1**^	0.934^1^	**0.003** ^**1**^
**Vegetables and fruits intake (days/week)**							
Less than 1 day/week 1–2 days/week 3–4 days/week 5–6 days/week Everyday	1.4 14.4 26.4 29.0 28.8	2.1 13.4 33.1 31.7 19.7	1.0 10.5 26.4 34.7 27.4	3.5 5.0 37.3 31.0 23.2			
**Vegetables and fruits/day** **(according to the average consumption of vegetables and fruits)**							
< 3 portions ≥ 3 and < 5 portions ≥ 5 portions	85.6 13.0 1.4	89.4 9.2 1.4	82.7 10.6 6.7	89.4 9.9 0.7	0.451^**2**^ **0.007 ³**	1.000^**2**^ 1.000 ³	0.079^2^ **0.006 ³**
**Intentions to eat vegetables and fruits next month**							
Same quantity Smaller quantity Larger quantity	63.5 6.3 30.2	67.6 4.9 27.5	63.5 4.8 31.7	60.6 6.3 33.1			
**Average consumption of dairy products (portions)**	1.20 (STD 1.19)	1.25 (STD 1.32)	1.23 (STD 1.05)	1.19 (STD 1.11)	0.734^1^	0.584^1^	0.717^1^
**Dairy products intake (days/week)**							
Less than 1 day/week 1–2 days/week 3–4 days/week 5–6 days/week Everyday	7.7 18.3 36.5 18.3 19.2	8.5 19.7 32.4 19.0 20.4	8.2 15.8 34.2 26.4 15.4	4.2 21.8 34.5 19.1 20.4			
**Dairy products/day** **(according to the average consumption of dairy products/day)**							
< 2 portions	79.3	77.5	78.8	81.0			
2–3 portions > 3 portions	14.9 5.8	14.8 7.7	16.4 4.8	12.7 6.3	1.000^**4**^	0.487^4^	0.624^4^
**Intentions to eat dairy products next month**							
Same quantity Smaller quantity Larger quantity	76.5 11.5 12.0	73.9 12.0 14.1	78.4 6.7 14.9	78.9 11.3 9.8			
**Low fat dairy consumption last week**							
Yes	51.0	38.7	50.0	37.3			
No I don’t know	26.9 22.1	33.1 28.2	30.8 19.2	35.2 27.5	0.644^5^	0.824^5^	0.090^5^
**Average consumption of soft drinks (cups)**	0.67 (STD 1.16)	0.83 (STD 1.33)	0.70 (STD 1.12)	0.84 (STD 1.24)	0.771^1^	0.946^1^	0.300^1^
**Soft drinks consumption (days/week)**							
Less than 1 day/week 1–2 days/week 3–4 days/week 5–6 days/week Everyday	21.6 44.2 22.6 5.8 5.8	21.1 45.1 15.5 9.2 9.1	24.0 40.9 25.9 4.4 4.8	27.5 35.9 21.8 6.3 8.5			
**Soft drinks/day** **(according to the average consumption of soft drinks/day )**							
< 1 cup 1-1.9 cups 2-2.9 cups 3-3.9 cups 4-4.9 cups ≥ 5 cups	81.7 10.1 2.4 1.0 1.4 3.4	74.6 13.5 4.9 2.1 0.0 4.9	79.3 13.0 3.8 0.5 0.0 3.4	73.2 12.0 8.5 2.1 1.4 2.8	0.603^6^ 1.000^7^ 0.454^8^ 0.581^9^	0.864^6^ 0.503^7^ 1.000^8^ 1.000^9^	0.184^6^ **0.033** ^**7**^ 0.286^8^ 0.676^9^
**Average consumption of sweets (portions)**	0.94 (STD 1.05)	1.16 (STD 1.29)	0.93 (STD 1.00)	1.10 (STD 1.13)	0.888¹	0.510 ¹	0.136 ¹
**Sweets consumption (days/week)**							
Less than 1 day/week 1–2 days/week 3–4 days/week 5–6 days/week Everyday	6.9 36.5 35.5 10.5 10.6	2.1 36.6 27.5 18.3 15.5	6.3 34.2 41.3 12.4 5.8	4.2 33.8 30.3 19.0 12.7			
**Sweets/day (according to the average consumption of sweets/day )**							
< 1 portion 1-1.9 portions 2-2.9 portions 3-3.9 portions 4-4.9 portions ≥ 5 portions	68.8 17.8 7.2 2.9 1.4 1.9	62.7 18.3 7.0 6.3 2.2 3.5	66.8 22.6 8.2 0.0 0.5 1.9	59.9 25.4 7.7 3.5 0.7 2.8	0.720^10^ 0.392^11^ 0.057^12^ 0.774^13^	0.659^10^ 0.327^11^ 0.118^12^ 0.453^13^	0.182^10^ 0.238^11^ **0.035** ^**12**^ 0.535^13^

Note: ^a^ Bold values represent statistical significance. ^*^ Differences between T2 vs. T1 were calculated using Dependent t test. ** Differences between I₂ vs. C₂ were calculated using Independent t test for continuous variables and Chi-test for categorical variables. ¹ Average consumption. ² <3 vs. ≥ 3 portions of vegetables and fruits, ³ <5 vs. ≥ 5 portions of vegetables and fruits. ^4^ <2 portions vs. ≥ 2 portions of dairy products. ^5^ Low fat dairy consumption last week, Yes vs. No. ^6^ <1 vs. ≥ 1 cups of soft drinks; ^7^ <2 vs. ≥ 2 cups of soft drinks; ^8^ <3 vs. ≥ 3 cups of soft drinks; ^9^ <4 vs. ≥ 4 cups of soft drinks. ^10^ <1 vs. ≥ 1 portions of sweets; ^11^ <2 vs. ≥ 2 portions of sweets.; ^12^ <3 vs. ≥ 3 portions of sweets; ^13^ <4 vs. ≥ 4 portions of sweets

Regarding dairy products, majority of children consumed them at least 3 days/week and less than 2 portions/day both at T1 and T2. Majority of children stated that they intended to consume the same amount the following month and in half cases in the Intervention groups, respectively more than a thirds cases in the Control groups, students reported that they consumed low fat dairy products the previous week.

Additionally, our findings suggest that more than half of schoolchildren consumed soft drinks between 1 and 4 days a week, from whom about a fifth in the Intervention groups and about a quarter in the Control groups served more than 1 cup/day.

With respect to sweets intake, our results indicate that majority of children consumed between 1 and 4 days/week, while about 20% in the experimental samples and around 30% in the Control groups served more than 1 portion/day.

When comparing T2 vs. T1 for those participating in both assessments (see Table 2), the Intervention group showed a significant increase in its average consumption of vegetables and fruits at T2 (I₂) compared to its baseline level (I₁). Furthermore, there was a notable rise in the proportion of individuals in I₂ who consumed at least 5 servings of vegetables and fruits per day, compared to I_1_ (*p* < 0.05), but also in the percentage of children eating ≥ 3 portions/day, although the result did not reach statistical significance. Control group at T2(C₂) reduced the average intake of fruits and vegetables compared to control group at T1 (C₁,) but results weren’t statistically significant. Also, the rate of students eating ≥ 3 servings/day remained steady, but it decreased the percentage of students eating at least 5 portions/day compared to T1. However, the difference did not reach the level of statistical significance.

Regarding dairy products, we noticed slight improvements at I_2_ for the average consumption, compared to I_1_, but with no statistical significance. Also, there was a modest increase in the percentage of children in the Intervention group who consumed ≥ 2 portions/day, but a decreasing trend in children consuming low fat dairy products the previous week, compared to T1, but results did not achieve statistical significance. No positive changes were noticed in the Control group (C₂) regarding the average intake of dairy products. Not only the proportion of children consuming more than 2 portions/ day decreased, but also the number of students eating low dairy fat products, compared to T1, however, with no statistical significance.

Additionally, there were no significant statistical differences in the average consumption of carbonated drinks between the follow-up and baseline measurements, although there was a slight increase in the average intake of carbonated drinks in the Intervention group at T2, compared to T1. Although the results did not reach statistical significance, when examining the Intervention group (I_2_), we observed an increase in the prevalence of children consuming ≥ 1 cup/day of sweetened beverages, but a decrease in the number of more than 2, 3 and 4 cups/day, compared to I_1_. On the other hand, within the Control group (C₂), there was an increase in the percentage of children consuming more than 1 and 2 cups/day compared to the first evaluation (C_1_), but a decrease in the prevalence of children consuming more than 3 and 4 cups, however with no statistical significance.

With respect to sweets intake, there were no significant changes in terms of average intake between follow-up and baseline in both groups. When examining the Intervention group, we observed an increase in the percentage of children consuming more than 1 portion of sweets per day compared to baseline, while the prevalence of participants consuming more than 2,3 and 4 portions per day decreased compared to T1, although results did not reach statistical significance. Within the Control group, at T2 we noticed an increase in the proportion of children consuming more than 1 portion per day, while the prevalence of students consuming more than 2, 3 and 4 portions per day decreased, but differences were not statistically significant.

### Self-reported eating habits during COVID-19 pandemic

Table 3 presents children’s perceived dietary changes during the COVID-19 pandemic.

**Table 3:** Self-reported eating habits during COVID-19 pandemic on the whole sample (*N* = 484)

	Intervention group				Control group				*p*-Value *
	Similar %	Increased %	Decreased %	I don’t know %	Similar %	Increased %	Decreased %	I don’t know %	
Fast food	27.3	7.3	56.0	9.4	25.5	14.2	46.7	13.6	**0.015** ^**1**^ **0.047** ^**2**^
Home cooked food	52.3	39.4	3.3	5.0	58.7	33.7	3.8	3.8	0.212^1^ 0.784^2^
Bought cooked food	33.0	9.3	42.0	15.7	34.2	9.3	37.5	19.0	0.972^1^ 0.327^2^
Potatoes	54.7	18.0	20.0	7.3	60.9	17.9	16.3	4.9	0.985^1^ 0.310^2^
Fresh vegetables and fruits	53.3	34.7	7.0	5.0	55.5	34.8	5.4	4.3	0.979^1^ 0.494^2^
Preserved/frozen vegetables and fruits	37.0	11.7	31.7	19.6	42.9	9.8	27.7	19.6	0.519^1^ 0.358^2^
Dairy products	58.0	25.3	10.0	6.7	57.6	25.5	12.0	4.9	0.958^1^ 0.499^2^
Meat	70.3	14.7	9.7	5.3	70.7	15.2	9.2	4.9	0.868^1^ 0.876^2^
Processed meat ( salami, sausages)	54.0	14.4	24.3	7.3	51.6	9.8	31.5	7.1	0.143^1^ 0.084^2^
Sweets (cakes, biscuits, chocolate, candies etc.)	46.0	17.0	29.7	7.3	40.2	18.5	35.3	6.0	0.678^1^ 0.194^2^
Chips, snacks	34.7	14.0	43.0	8.3	39.7	14.6	37.5	8.2	0.836^1^ 0.232^2^
Soft drinks	35.0	10.7	44.7	9.6	41.3	12.0	39.1	7.6	0.661^1^ 0.231^2^
Energy drinks	26.7	9.7	38.6	25.0	30.4	7.6	40.8	21.2	0.439^1^ 0.647^2^
Rice, pasta	52.7	19.7	18.6	9.0	54.9	23.4	12.5	9.2	0.332^1^ 0.074^2^
Bread	59.7	18.0	16.3	6.0	60.3	18.0	17.4	4.3	0.985^1^ 0.762^2^

Note: Chi-test results

* Bold values represent statistical significance;

¹ Differences between *Increased* vs. *Rest of it* in I₂ vs. C₂ groups;

² Differences between *Decreased* vs. *Rest of it* in I₂ vs. C₂ groups

For children in the Intervention group the highest percentages of reported similar food consumption was for intake of meat (70.3%), bread (59.7%), dairy products (58.0%), followed by potatoes, processed meat, fresh vegetables and fruits, rice and pasta, home cooked food (a bit more than half) and sweets (almost half). On the other hand, 39.4% of them increased their level of home cooked food, 34.7% of them of fresh vegetables and fruits, 25.3% of them of dairy products. Notably, there was a decrease in the consumption of unhealthy food items like fast food for 56%, soft drinks for 44.7%, chips/snacks for 43%, sweets for 29.7% along with a decrease in the consumption of purchased cooked food for 42%.

From highest to lowest, children in the Control group reported a similar intake during pandemic for meat (70.7%), potatoes (60.9%) and bread (60.3%), followed by home cooked food, dairy products, fresh vegetables and fruits, rice and pasta and processed meat (more than half), sweets and soft drinks (around 40%). On the other hand, around one third of children increased their consumption levels of fresh vegetables and fruits (34.8%) and home cooked food (33.7%), while around 4 out of 10 stated they decreased the level of fast food (46.7%) and of soft drinks (39.1%), followed by chips and snacks (37.5%), bought cooked food (37.5%), sweets (35.3%) and processed meat (31.5%).

### The impact of the school-based health program on dietary habits

According to data from Table 1, at baseline, there were no statistically significant differences between Intervention and Control groups in terms of average consumption and portions of food for each item included.

However, at T2, notable improvements were observed (see Table 2). Children in the Intervention group increased their average intake of fruits and vegetables compared to the Control group and notably, they consumed more frequently ≥ 5 portions/day of fruits and vegetables than the Control group (*p* < 0.05). Additionally, there were slight improvements in the Intervention group concerning the average consumption of dairies compared to the Control group, although results did not reach statistical levels. Moreover, students of the experimental group consumed ≥ 2 servings/day of dairy more frequently but also low-fat dairy products than in Control group, but differences weren’t statistically significant. There were no significant statistical differences in the average consumption of carbonated drinks and sweets between I_2_ vs. C_2_. We also noticed, children in the Control group reported a more frequent consumption of ≥ 2 portions of drinks compared to those in the Intervention group, results which were statistically significant.

Regarding sweets intake, there was a significant difference in the consumption of more than 3 portions per day, with children in the Intervention group reporting a less frequent consumption compared to the Control group.

Regarding children’s perceived eating habits during pandemics, when comparing the Intervention and Control groups, several differences were observed (Table 3). On one hand, there was a significant increase in the percentage of students in the experimental sample who reported that they decreased their consumption of fast food compared to those in the Control group. On the other hand, although results did not reach the statistical significance, we noticed that the Intervention group, compared to the Control group, reported a greater decrease in the consumption of unhealthy food items, such as fast food (56.0% vs. 46.7%), chips and snacks (43% vs. 37.5%), soft drinks (44.7% vs. 39.1%), but also purchased cooked food (42.0% vs. 37.5%), while the Control group showed a larger decrease in the consumption of energy drinks compared to the Intervention group (40.8% vs. 38.6%). Furthermore, we noticed that a higher proportion of children in the Intervention group reported a similar intake of dairy products, processed meat, and sweets compared to the Control group, whereas the Control group had a higher proportion of children reporting a similar intake for home-cooked food, potatoes, fresh vegetables and fruits, as well as preserved/frozen vegetables and fruits.

### Factors which influence dietary habits

At T1, younger age was identified as a factor associated with increased consumption of fruits and vegetables. Concerning dairy products, younger children, especially boys, tended to consume larger amounts. Students who intended to lose weight in the previous year were more likely to reduce their intake of dairy products. High consumption of carbonated drinks was associated with older age, being a boy and intentions to gain weight. On the other hand, intentions to lose weight and a higher BMI were associated with a trend towards a reduction in soft drinks. Moreover, older children and those who had tried to gain weight in the previous year were more likely to increase the amounts of sweets, while children who attempted to lose weight tended to reduce their intake (see Table 4).

**Table 4:** Factors associated with dietary habits at baseline (*N* = 880)

	Average consumption of fruits and vegetables/day	Average consumption of dairy prod/day	Average consumption of carbonated drinks/day	Average consumption of sweets/day
	ß (95%CI)	ß (95%CI)	ß (95%CI)	ß (95%CI)
**Age**	-0.13 (-0.21, -0.07)***	-0.09 (-0.17, 0.02)**	0.07 (0.00, 0.15)*	0.07 (0.01, 0.16)*
**Gender** Male (vs. Female)	0.05 (-0.03, 0.29)	0.10 (0.09, 0.43)**	0.09 (0.07, 0.41)**	0.02 (-0.11, 0.22)
**Participants** Intervention (vs. Control)	0.01 (-0.13, 0.20)	0.02 (-0.11, 0.23)	-0.01 (-0.21, 0.13)	0.00 (-0.16, 0.17)
**Attempts to lose weight in the last year** Yes (vs. No)	0.00 (-0.18, 0.15)	-0.07 (-0.36, 0.01)*	-0.10 (-0.45, -0.10)**	-0.13 (-0.51, -0.17)***
**Attempts to gain weight in the last year** Yes (vs. No)	0.01 (-0.19, 0.28)	0.01 (-0.18, 0.30)	0.19 (0.47, 0.96)***	0.20 (0.52, 0.99)***
**BMI**	-0.02 (-0.02, 0.01)	-0.06 (-0.04, 0.00)	-0.06 (-0.04, 0.00)*	-0.05 (-0.03, 0.00)

* *p* < 0.05; ** *p* < 0.01; *** *p* < 0.001

As presented in Table 5, the cross-sectional data show that at T2, younger age and experimental group were positively associated with a higher fruit and vegetable intake, boys were more likely to consume larger amounts of carbonated drinks, children with weight loss intentions in the previous year tended to decrease their consumption of sweets, while those who intended to gain weight were linked to an increase in their consumption of sweets.

**Table 5:** Factors associated with dietary habits at follow-up on the whole sample of children (*N* = 484)

	Average consumption of fruits and vegetables/day	Average consumption of dairy products/day	Average consumption of carbonated drinks/day	Average consumption of sweets/day
	ß (95%CI)	ß (95%CI)	ß (95%CI)	ß (95%CI)
**Age**	-0.15 (-0.33, -0.06)**	0.07 (-0.03, 0.20)	0.06 (-0.05, 0.21)	0.07 (-0.03, 0.21)
**Gender** Male (vs. Female)	-0.02 (-0.25, 0.15)	0.05 (-0.06, 0.30)	0.12 (0.07, 0.50)**	-0.04 (-0.29, 0.09)
**Participants** Intervention (vs. Control)	0.10 (0.02, 0.44)*	0.02 (-0.13, 0.25)	-0.02 (-0.28, 0.15)	-0.03 (-0.27, 0.13)
**Attempts to lose weight in the last year** Yes (vs. No)	0.04 (-0.10, 0.31)	-0.08 (-0.37, 0.01)	-0.00 (-0.22, 0.21)	-0.11 (-0.46, -0.05)*
**Attempts to gain weight in the last year** Yes (vs. No)	-0.05 (-0.51, 0.13)	0.05 (-0.12, 0.48)	0.08 (-0.02, 0.66)	0.16 (0.26, 0.89)***

* *p* < 0.05; ** *p* < 0.01; *** *p* < 0.001

Table 6 presents predictive factors of dietary habits for children who participated in both assessments. Predictors of fruits and vegetables intake at T2 has several characteristics at T1: younger age, participation in the Intervention group and increased consumption of vegetables and fruits in T1. Similarly, children who consumed more dairy products at baseline predicted increased consumption of dairies at T2. Boys, children who reported intending to gain weight in the previous year at the first assessment and those with an increased intake of carbonated beverages in baseline predicted greater amounts of carbonated drinks. Students who reported weight loss intentions in the previous year at T1, predicted a decrease in sweets intake at follow-up. Children who reported trying to gain weight in the previous year at baseline, as well as those were already consuming more sweets at T1 predicted increased consumption of sweets at T2.

**Table 6:** Predictive factors of dietary habits for children who participated in both assessments (*N* = 350)

	Average consumption of fruits and vegetables/day	Average consumption of dairy products/day	Average consumption of carbonated drinks/day	Average consumption of sweets/day
	ß (95%CI)	ß (95%CI)	ß (95%CI)	ß (95%CI)
**Age**	-0.15 (-0.33; -0.06)**	0.07 (-0.03; 0.20)	0.06 (-0.05; 0.21)	0.07 (-0.03; 0.21)
**Gender** Male (vs. Female)	-0.00 (-0.25; 0.24)	0.02 (-0.18; 0.27)	0.15 (0.12; 0.61)**	0.04 (-0.13; 0.31)
**Participants** Intervention (vs. Control)	0.16 (-0.13; 0.63)**	0.01 (-0.18; 0.27)	-0.05 (-0.38; 0.11)	-0.08 (-0.39; 0.05)
**Attempts to lose weight in the last year** Yes (vs. No)	0.06 (-0.0; 0.41)	-0.02 (-0.28; 0.18)	-0.03 (-0.32; 0.17)	-0.10 (-0.45; -0.00)*
**Attempts to gain weight in the last year** Yes (vs. No)	-0.03 (-0.48; 0.24)	0.03 (-0.21; 0.45)	0.16 (0.19; 0.91)**	0.23 (0.39; 1.03)***
**Average intake of fruits and vegetables at T1**	0.27 (0.17, 0.38)***	-	-	-
**Average intake of dairy products at T1**	-	0.29 (0.16, 0.34)***	-	-
**Average intake of carbonated drinks/day at T1**	-	-	0.34 (0.23, 0.42)***	-
**Average intake of sweets/day at T1**	-	-	-	0.39 (0.27, 0.45)***

* *p* < 0.05; ** *p* < 0.01; *** *p* < 0.001

## Discussion

To our knowledge, this is the first longitudinal study conducted in Romania that provides data regarding changes in dietary patterns prior and during COVID-19 pandemic among Romanian school children.

Our initial findings offer an overview of the children’s behaviors in terms of food habits before the onset of the pandemic. Regarding the vegetables and fruits intake, our outcomes align with results from other studies conducted on both Romanian and international populations [59], validating the inadequate consumption of fruits and vegetables among students. In our study, the average intake was 1.57 in the Intervention sample and 1.53 portions/day in the Control sample, which was lower than WHO recommendations of 5 portions/day [60]. On smaller samples, two studies conducted in 2011 in Cluj-Napoca, a developed city in Romania, revealed that half of the students consumed fruits daily, while one-third of them consumed vegetables every day [57, 61]. 

In relation to dairy products, our research revealed similar concerning results. We noticed that the consumption of dairy among children was inappropriate. Less than 20% of students in both groups stated they consumed them daily, and even fewer (14.3% respectively 12.7%) declared they served between 2 and 3 portions/day. Our results are consistent with several other studies that showed young people, especially adolescents, fail to meet the recommended daily intakes [48, 62, 63] of 2–3 servings/day for this young population [63]. A Canadian study showed that in 2015, most teenagers under consumed dairy products and only 26% met the recommended guidelines [64]. Similarly, a study in the Netherlands reported that a third of children and adolescents in their sample did not consume milk [65]. Among factors that influence children’s dairy foods consumption, literature cited gender, parental dietary behaviors, replacing milk with other beverages, and the quality of the diet [48]. Higher intake of milk-based products was found to be associated with increased consumption of cereals, fruits and vegetables [65], eating fish, use of olive oil [63], suggesting that dairy products represent a marker for healthy eating habits [66]. Furthermore, dairy products have been associated with lower odds of unhealthy eating, such as regular sweets [63], but also small amounts of non-alcoholic beverages, especially soft drinks and tea and coffee [65], which may confirm that competing food items may substitute dairy products [67].

Regarding carbonated beverages and sweets, we noticed an increased consumption among children. In line with other studies, results of the current research show that children’s diet quality has worsened, as youth consume more energy-dense foods [68, 69]. Children have replaced healthy eating habits (such as fruits and vegetables and milk) with nutrient-poor diets that deliver empty calories [26]. The latest report of the 2021/2022 Health Behavior Survey in School-aged Children (HBSC) provides an insight into the eating behaviors of adolescents in 44 countries and regions in Europe, Central Asia and Canada. According to it, there is a decrease in children’s healthy eating habits, but an increase in unhealthy choices [70]. Data showed that 38% of children eat fruit or vegetables every day and more than half of them do not consume either fruit or vegetables daily. On the other hand, sweets and soft drinks are highly consumed. Daily sugary drinks intake saw a small decline since the last report in 2018, but even so, it still stands at 15% of adolescents. When it comes to sweets and chocolate, one in four teenagers eat them daily. Moreover, these figures show differences by age and gender: for fruits, consumption decreased with age in both sexes, the highest prevalence was seen in 11 years old girls (47%) and the lowest in 15-years old boys (32%). Similarly, 40% of 11 years old children ate vegetables daily, while 36% of 15 years-old children served them every day. Girls consumed more often sweets and chocolate than boys (28% vs. 23%) and have increased their intake with age. The biggest gender disparities across all age groups were noticed in Romania. In contrast, consumption of soft drinks in boys remained higher than in girls ( 16% versus 14% for girls) [71].

Our results are similar to available data that suggests transition from childhood to adulthood is associated with a decline in healthy dietary habits: while there is a reduction of fruits, vegetables and dairy products intake, there is an upper consumption of sweets and soft drinks [72, 73]. Moreover, these patterns extend to other age groups, indicating that unhealthy dietary habits developed during childhood can endure into adulthood [54]. Another study conducted on the population of Cluj-Napoca, which analysed two waves between 2003 and 2016 among university students, showed that the consumption of vegetables and fruits remained low but stable. The majority of participants reported consuming fruits and vegetables at least once a week, while less than 20% consumed them daily. Also, the proportion of students consuming dairy products daily decreased from half at T1 to one third at T2. In both waves, approximately one out of five students reported consuming sweets on a daily basis [74].

Moreover, our results from the second assessment support these hypotheses. At follow-up, on the entire sample of students, we noticed some changes in children’s dietary behaviors. Consumption of vegetables and fruits remained consistent with baseline, while dairy products showed a slightly decreasing intake. Regarding consumption of carbonated drinks and sweets, in both Intervention and Control groups, majority of children consumed them in large quantities. These results emphasize the need for consistent efforts to promote healthy eating habits among pupils in Romania, taking into account various factors that could influence them. The follow-up assessment overlapped with the COVID-19 pandemic, a time when people suddenly changed their routines. Food habits were not “immune” to the COVID-19 crisis as well.

From a longitudinal perspective, the analysis revealed, however, a statistically significant increase in the proportion of individuals in I₂ who consumed at least 5 servings of vegetables and fruits per day compared to I₁ and a substantial increase in the average intake of fruits and vegetables at I₂. Nevertheless, many children still did not reach the recommended daily intake. A previous study carried out in Romania in 2021 during pandemic, among a smaller sample consisted of 285 students, showed that children avoided fruits and vegetables altogether, but mostly the vegetables [37]. Our results are particularly concerning, as the study revealed that students are not fully aware of these unhealthy habits, with many students expressing their intention to maintain a similar level of consumption in the upcoming month. Regarding dairy products, at I₂, the percentage of children who consumed daily milk-based products decreased to 15.4% (from 19.2% at T1). Similar to fruits and vegetables, most children intended to consume the same amount. In terms of soft drinks and sweets, more than half of students consumed them between one to four days per week. In both samples, a significant proportion of children exceeded the recommended guidelines.

Our study highlighted changes not only according to the data declared by the students, but they themselves also perceived differences. Based on children’s self-reported perceptions on dietary patterns during remote education period, our study revealed positive changes towards a healthy diet. Children in the Intervention group decreased consumption of comfort food such as fast food and soft drinks, whereas consumption of various food categories such as potatoes, fresh vegetables and fruits, dairy products, meat, processed meat, rice and pasta, and bread remained relatively stable. Conversely, children in the Control group reported a decrease of fast food, soft drinks, chips and snacks, sweets, while the level of meat, potatoes, bread, home cooked food, dairy products, fresh vegetables and fruits, rice and pasta were similar. These outcomes could be attributed to what So Young Kim et al. emphasized in their research that health-seeking behavior might reduce consumption of unhealthy food items. During pandemic, people were repeatedly exposed to health campaigns and education on quarantine measures through mass media [10]. Similarly, a review of Jayawardena and authors, suggested that a balanced nutrition helps maintain the immune system which is crucial for limiting viral contagion, particularly among vulnerable population [75]. Comparing the results obtained in other studies conducted during similar periods on adolescents, we observe certain trends among the Romanian and European population. While some studies reported an increase in the consumption of sweets during the health crisis [76, 77], (in contrast to the results presented in this research), in others, the intake of carbonated beverages decreased [78, 79], while the consumption of fruits and vegetables increased [78, 80].

These assumptions align with the following results we obtained. In both samples, children reported they decreased consumption of purchased cooked-food, while the intake of home-cooked food remained relatively similar. This latter aspect may be explained by the fact that children spend more time together with their family preparing dishes. Home cooking creates a supportive setting where children can cultivate healthy habits. It enables nutritious discussions and provides opportunities for parents to be role models in promoting healthy eating behavior [10, 81].

Schools are widely acknowledged as promising settings for public health interventions due to their extensive reach among children and youth. With their formal role in providing health and physical education, they have the potential to effectively promote and implement initiatives aimed at improving public health [82]. Health education programs typically encompass a wide range of targets, including nutrition knowledge, physical activity, fruits and vegetable intake, sugar consumption [43]. While majority of interventions focus on improving children’s knowledge towards a qualitive diet, others target the availability of fruits and vegetables within the school environment [43], or involve parents in adoption of healthy eating habits [9, 16, 43].

The findings from our research indicated that the educational program had a positive impact on participants. Starting from the same dietary habits at T1 among the Intervention group and Control group, the longitudinal data revealed statistically significant improvements in certain aspects. Firstly, students in the Intervention group increased both the average fruits and vegetables daily intake and the prevalence for serving ≥ 5 portions/day, compared to the Control group. Similarly, another study showed moderate effects of the intervention program. The PROFRUVE study indicated an increase of 0.31 portions of fruits and 0.14 servings of vegetables. These improvements persisted twelve months follow-up, post intervention [83]. Secondly, our findings showed children in the Intervention group drank less than ≥ 2 cups per day of carbonated beverages than the Control group.

Additionally, regarding children’s perceived dietary during pandemics, when comparing the Intervention and Control groups, we noticed interesting results. Although the differences were not statistically significant, except for fast food, the Intervention group showed a greater decrease in consumption of chips and snacks, soft drinks, fast food and purchased cooked food and a similar intake of dairy products, processed meat, and sweets compared to the Control group. Overall, although the results lean towards healthier changes in the Intervention group than in the Control group, additional factors such as individual preferences and home environment could influence the differences between the two groups and should be explored in further research.

Our favourable outcomes observed in this study can be attributed to the implementation of an evidence-based approach in developing our nutrition and health educational curriculum. For instance, we involved teachers serve as role models for healthy behaviors. To address the limitations faced by teachers in implementing nutrition education programs, as highlighted by De Vlieger et al. [9], we ensured that teachers received adequate assistance to effectively teach their students about nutrition by providing them with supportive and printed materials. Furthermore, we implemented interactive games designed to empower children to make healthy choices. Moreover, our multi-component approach, which combined curriculum-based learning with provision of posters, take-home messages, student’s book, newsletters for parents, and fruits and vegetable tastings, likely contributed to children’s positive overall attitude towards healthy habits. However, it’s important to acknowledge that the evaluation of this intervention occurred more than a year later. Despite this extended period, in which children went through individual and environmental factors along with the challenges generated by COVID-19 as well as the lack of any subsequent educational interventions, we still noticed positive changes in children’s eating patterns.

Last, but not least, our study identified factors associated with children’s dietary habits. At baseline, younger age was associated with an increased consumption of fruits and vegetables, dairy products, whereas older age with elevated soft drinks and sweets intake. These findings are consistent with previous research and could be related to the fact that older children are more independent in their choices and freer in purchasing these foods than their younger counterparts [39, 84]. Additionally, boys tended to consume more dairy products and carbonated drinks. These outcomes correspond with other studies that showed boys consumed larger amounts of milk and more sweets and sugar-sweetened beverages than girls [23, 49, 85]. Louie et al. in their communication highlighted children’s misconception that dairy products are fattening. This could explain why girls may consume inadequate amounts of dairy products, as they are more concerned with their body image and more likely to engage in dieting, including reduction dairy [62]. On the other hand, girls are thought to be more likely to rate healthy behaviors than boys [86]. Our finding supports these ideas, as children in our sample who intended to lose weight the previous year were more likely to decrease the consumption of dairy, soft drinks and sweets, while children who aimed to gain weight tended to drink more soft drinks and eat more sweets. Moreover, children with a low BMI tended to consume a higher quantity of carbonated beverages.

At follow-up, on the whole sample of children, younger age, participation in the educational program were linked to an increase intake of fruits and vegetables. Boys were more likely to consume more soft drinks and individuals who intended to gain weight in the preceding year tended to have a higher intake of sweets.

Finally, according to longitudinal available data, pupils’ dietary habits from T1 were predictors at T2. Consequently, children who initially consumed limited amounts of fruits, vegetables, dairy, carbonated beverages and sweets continued this trend at follow up.

Our study is subject of several strengths and limitations. One of the strengths is that it was designed as a longitudinal study, allowing us to assess two distinct periods of time, one of which coincided with the atypical situation of the COVID-19 period. This design allowed us to observe changes in diet amidst a pandemic. Additionally, to the best of our knowledge, this is the first study in Romania that provides longitudinal data on these changes, adding to the existing literature.

However, there are limitations to our study too. One limitation is that our baseline cohort consisted in urban settings of two counties of Romania. Therefore, it is important to acknowledge that the generalization of these findings to the entire country may be limited. Furthermore, the cohort of children who were assessed at both T1 and T2 consisted of only 39.7% participants. This was mainly due to the safety measures imposed during the COVID-19 crisis, which made it difficult for us to conduct the second assessment among students. Moreover, the transition to online was a challenge for us, as we had to adapt the data collection method. To reach students we relied exclusively on teachers to distribute the questionnaire. The use of digital survey might have affected the data collection process as some students might have hesitated in providing their names. Furthermore, we did not assess others factors that could have influenced the dietary consumption, such as food preference, taste or income. To assess eating habits, we used a food frequency questionnaire, which has some limitations compared to a food tracking diary or the 24-hour recall. Finally, similar with many studies in the field, our assessment was based solely on children’s statements, parents were not involved in order to guide and assist the filling of the questionnaire by children.

## Conclusions

We implemented a school-based educational program and analysed changes in children’s eating behaviors in a longitudinal study. Although the second assessment took place over a year later, a time when children inevitably go through psycho-social and environmental changes, and to which, in addition, the challenges generate by the COVID-19 crisis were added, our intervention had success by improving healthier habits in children. These positive results highlight the crucial role of health education and further emphasize the ongoing need to incorporate such initiatives into regular activities and actions that encourage healthy eating and overall well-being.
